# Collagen Type II-Targeting Lentiviral Gene Therapy for Mucopolysaccharidosis IVA

**DOI:** 10.3390/cimb48010042

**Published:** 2025-12-27

**Authors:** Betul Celik, Sampurna Saikia, Shaukat Khan, Krishna Sai Musini, Shunji Tomatsu

**Affiliations:** 1 Nemours Children’s Health, 1600 Rockland Rd., Wilmington, DE 19803, USA; betul.celik@nemours.org (B.C.); ssaikia@udel.edu (S.S.); shaukat.khan@nemours.org (S.K.); krishnasai.musini@nemours.org (K.S.M.); 2 Department of Biological Sciences, University of Delaware, Newark, DE 19716, USA; 3 Department of Pediatrics, Graduate School of Medicine, Gifu University, Gifu 501-1193, Japan; 4 Department of Pediatrics, Thomas Jefferson University, Philadelphia, PA 19107, USA

**Keywords:** lentiviral gene therapy, CBh, COL2A1, collagen-type II targeting, WYRGRL peptide, MPS IVA, intravenous and intraarticular injection, newborn treatment

## Abstract

Mucopolysaccharidosis (MPS IVA) is caused by pathogenic variations in the *GALNS* gene, leading to the accumulation of glycosaminoglycans in tissues and causing progressive skeletal lesions. While conventional lentiviral vectors (LVs) provide long-term stable expression, they do not deliver therapeutic levels to bone and cartilage. We hypothesized that engineering the LV envelope with a collagen type II-targeting peptide (WYRGRL) increases the binding affinity of the LVs for bone and cartilage. These modified vectors carrying the CBh and COL2A1 promoters delivered the *GALNS* gene to MPS IVA newborn mice via intravenous (IV) or intraarticular (IA) administration. The peptide-modified LVs exhibited markedly increased uptake in the liver when administered IV, but lower enzyme activity than that of the conventional vector. The modified WYRGRL-LV-COL2A1 vector elevated GALNS activity in other tissues, suggesting systemic benefits. When administered IA, the modified vectors showed potential for local treatment due to the WYRGRL peptide-mediated uptake. Additionally, there was a reduction in keratan sulfate glycosaminoglycan levels in plasma and tissues, indicating that this peptide can be a suitable candidate for disease modification. These findings pave the way for further preclinical and clinical studies, offering new possibilities for the development of targeted therapies for skeletal diseases.

## 1. Introduction

Pathogenic variations in the *GALNS* gene result in the accumulation of GAGs, including keratan sulfate (KS) and chondroitin 6-sulfate (C6S), within lysosomes, leading to one of the lysosomal storage disorders, MPS IVA (also known as Morquio A syndrome). MPS IVA is a progressive systemic skeletal dysplasia, although affecting multiple tissues, including the cornea, trachea, bone, cartilage, ligaments, other connective tissues, heart, and lungs [1].

The buildup of these GAGs in the avascular growth plate of MPS IVA patients significantly contributes to growth impairment and skeletal dysplasia. There is no evidence that current ERT affects bone growth and skeletal lesions in MPS IVA patients [2,3,4,5,6]. Chondrocytes from MPS IVA patients treated with ERT remain fully vacuolated with storage materials [2,7]. In the MPS IVA mouse model, ERT was tested on newborns and did not improve bone pathology [8]. AAV is another promising approach; however, it creates a strong immune response and cannot be repeated. Additionally, when the AAV vector is administered to newborns, there is a risk of losing the *GALNS* gene expression (dilution effect) due to growth and development. AAV gene therapy consistently demonstrated high expression of GALNS enzymes in the liver, resulting in partial correction of bone pathology; however, it did not fully restore the columnar structure and vacuolated chondrocytes [9]. Conventional LVs are integrated into the genome, providing stable, long-term expression; however, achieving a therapeutic level in bone remains an unmet challenge. Therefore, no single, long-term, effective treatment has been found to ameliorate disease progression in this skeletal dysplasia. Furthermore, the engineering of the viral envelope has become a popular approach in LV gene therapy, particularly to prevent immune reactions [10,11,12].

Targeting the growth plate is crucial for bone development in many bone diseases. However, this area is avascular and difficult to access through blood vessels. The growth plate region consists of a layer of different chondrocytes. The ECM of this region includes various types of collagens, such as types I, II, XI, and X [13,14,15,16]. Type II collagen serves as a biomarker of the chondrocyte phenotype in the proliferative zone, while type X collagen indicates late-stage chondrocytes in the hypertrophic zone [17]. Type II collagen is mainly found in the ECM of cartilage, partially in the vitreous gel, and in intervertebral discs [16]. Since type II collagens account for about 90 to 95% wth plates, several other collagens are closely associated with the α1 chain of type II collagen bundles [18]. The effectiveness of the WYRGRL peptide has been demonstrated in mice, rats, and pigs with osteoarthritis, particularly through conjugation to nanoparticles, modification of mesenchymal stem cells to produce extracellular vesicles or exosomes, and hydrogels [19,20,21,22,23,24,25,26]. Targeting type II collagen with LVs pseudotyped with the WYRGRL-VSVG ligand at an early stage might enhance LV uptake, potentially improving disease outcomes [26,27]. LVs may also be incorporated into the ECM as a source for chondrocyte uptake, as shown in previous nanoparticle studies [24,28,29]. Furthermore, to maximize therapeutic effects in cartilage chondrocytes—a challenging avascular region—targeting the deep zone of the cartilage layer is essential. The WYRGRL peptide reaches this zone via cartilage-specific interactions with collagen type II [24]. These are vital aspects of using a collagen-targeting peptide to reach chondrocytes that are impaired in MPS IVA disease. Our goal was to develop an effective bone-targeting treatment using the novel collagen type II-binding WYRGRL-VSVG-engineered LV to target bone and cartilage lesions in MPS IVA mice. We hypothesized that adding collagen-targeting ligands to the LV envelope would increase the binding affinity to bone and cartilage, thereby enhancing gene delivery and tissue targeting, ultimately alleviating bone lesions in MPS IVA mice. The new LV system may offer advantages, including higher enzyme activity in bone and cartilage, which could improve bone pathology and enhance patients’ quality of life in MPS IVA patients. This strategy might also benefit other severe skeletal dysplasias.

## 2. Materials and Methods

### 2.1. Mouse Maintenance

We used MPS IVA KO mice (*Galns^−^*^/^*^−^*, MKC2) [30]. The age-matched male (wild-type and untreated) mice were used as controls. The colony was housed in a pathogen-free facility on a 12-h light/dark cycle. All mouse care and handling procedures were by the rules of the Institutional Animal Care and Use Committee (IACUC) of Nemours Children’s Health, Delaware Valley (protocol code RSP20-12482-005 and 12 March 2025 of approval)—“Gene delivery for Morquio A by lentiviral vectors.”

### 2.2. Construction of LVs

**Figure 1:**
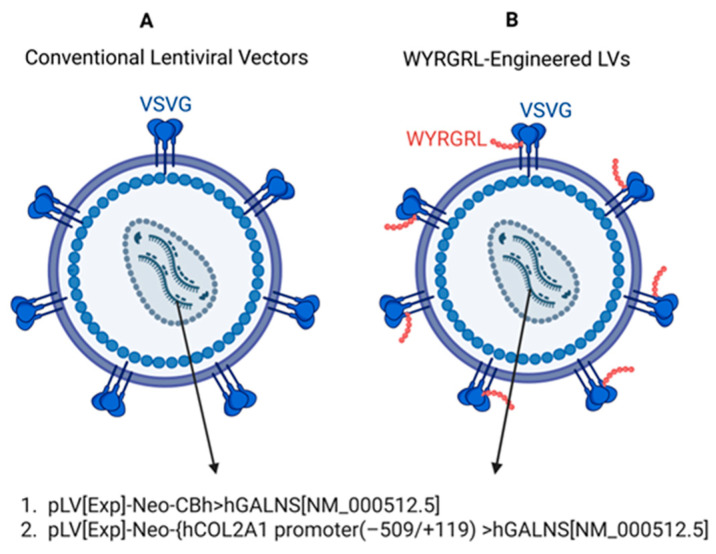
LVs delivering native *GALNS* genes under CBh and COL2A1 promoters. (**A**). Conventional LVs and (**B**). WYRGRL-engineered LV.

We designed LVs under ubiquitous CBh and cartilage-specific transcription factors binding COL2A1 promoters driving the expression of the native human *GALNS* gene, as reported previously [30]; the final expression constructs were pLV[Exp]-Neo-CBh>hGALNS[NM_000512.5] and pLV[Exp]-Neo-{hCOL2A1 promoter(−509/+119)}>hGALNS[NM_000512.5]. To increase the uptake of LVs by the bone and cartilage cells, we engineered the VSVG pseudotyped LV envelope. Briefly, the WYRGRL collagen type II targeting peptide sequence was inserted into the N-terminal region of the VSVG ligand. Position of WYRGRL on the VSVG encoding plasmid is 1297-1314 after the start codon, and the length is 18 nucleotides. The entire WYRGRL/VSVG position is 1297-2847, and the length of which is 1554 nucleotides. Then, WYRGRL-VSVG pseudotyped LVs were generated in HEK293 cells (Figure 1).

### 2.3. Administration of LVs and Tissue Collection

**Figure 2:**
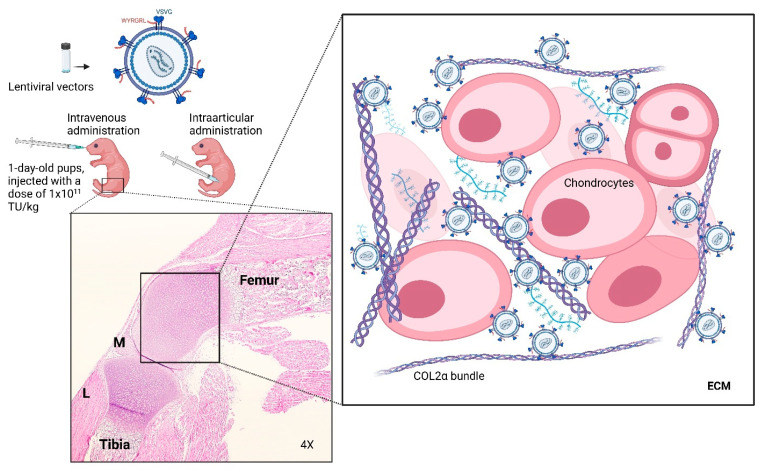
IV or IA administration of LVs into 1-day-old male MPS IVA mice. L: Ligament, M: Meniscus. Visualized at 40× magnification. LVs were incorporated into the ECM and taken up by either collagen type II or LDL receptors.

The effects of LVGT in MPS IVA mice were evaluated in terms of enzyme activity, GAG levels, pathology, and immune responses to the transgene product. MKC2 mice received 1 × 10^11^ TU/kg *GALNS* LVs, as determined in our previous study [30]. Treatments were administered intravenously via a superficial temporal vein in newborns or intraarticularly through the medial cavities between the femur and tibia (Figure 2). As controls, untreated MKC2 and wild-type mice of the same age received saline. At 16 weeks, five mice per group were euthanized, and tissues—including WBCs, BMCs, brain, heart, lung, liver, kidney, spleen, muscle, femur–tibia, arm, trachea, and lymphoid tissues such as the thymus and lymph nodes—were collected. Blood samples and WBCs were taken at baseline and every two weeks (Figure 2).

### 2.4. Tissue Homogenization and GALNS Enzyme Assay

The GALNS enzyme activity was carefully measured in plasma and tissue extracts using a 4-methylumbelliferone (4-MU) assay (Melford Laboratories Ltd., Suffolk, UK). Briefly, tissue was dissected and immediately homogenized with a Bead Mill Homogenizer (OMNI International, Kennesaw, GA, USA) in homogenization buffer (25 mM Tris–HCl, pH 7.2, 1 mM PMSF). Homogenates were centrifuged for 30 min at 4 °C, and the supernatant was transferred to a new tube. The tissue supernatants or plasma samples underwent the 4-MU enzyme assay, where 2 μL of tissue supernatants were incubated with 9 μL of 22 mM 4-methylumbelliferyl-β-galactopyranoside-6-sulfate (Research Products International, Mount Prospect, IL, USA) at 37 °C for 16 h. The next day, 2 μL of 10 mg/mL β-galactosidase from Aspergillus oryzae (Sigma-Aldrich, St. Louis, MO, USA) was added to the reaction and incubated for 1 h at 37 °C. The reaction was stopped with 1 M glycine buffer (pH 10.5, adjusted with NaOH). We used the FLUOstar Omega plate reader (BMG LABTECH Inc., Cary, NC, USA) to measure enzyme activity at an excitation wavelength of 336 nm and an emission wavelength of 450 nm. The activity was expressed as nanomoles of 4-methylumbelliferone released per hour per milligram of protein (nmol/h/mg). Protein concentrations were determined using a PierceTM BCA assay kit (Thermo Fisher Scientific #23225, Waltham, MA, USA).

### 2.5. Evaluation of GAGs

We confirmed the reduction of accumulated GAGs using LC-MS/MS, as described in the previously published protocol [31,32].

### 2.6. Biodistribution of LVs

The biodistribution of LVs in MKC2 mice was examined in all tissues collected at 16 weeks post-infusion. DNA was extracted from tissue samples using the Qiagen Gentra Puregene Tissue Kit (Qiagen, Germantown, MD, USA) and digested with Proteinase K at 55 °C. After RNase treatment, the DNA was resuspended in Tris-EDTA buffer, and its concentration was measured. The VCN quantification was performed using ddPCR (ThermoFisher QuantStudio Absolute Q, Waltham, MA, USA) as a single-plex assay (LV and Tfrc on two separate chips) with primers (Integrated DNA Technologies, San Diego, CA, USA) specific to the LV vector psi (Ψ) gene, producing an 82-bp PCR fragment (Table S1). The reaction was prepared with the QuantStudio™ Absolute Q™ MAP16 Plate Kit and Master Mix (ThermoFisher #A53301, Waltham, MA, USA). Quantification was calculated using the formula: (LV cps/µL × DNA dilution)/(Tfrc cps/µL/2).

### 2.7. GALNS mRNA Expression Analysis

To detect GALNS transcripts in the liver, spleen, lung, ear, and bone at 16 weeks post-infusion, total RNA was extracted from each mouse tissue using the QiaAmp RNA kit (Qiagen, Germantown, MD, USA) according to the manufacturer’s instructions. A TaqMan gene expression assay was used for qPCR. GAPDH was used to normalize the input. Each experiment was conducted in duplicate. Data were analyzed using the comparative Ct method.

### 2.8. Systemic Toxicity Analysis

Peripheral blood samples, collected 16 weeks after infusion, were examined for LVGT toxicity by measuring ALT (BioAssaySystems#EALT-100, Hayward, CA, USA) and AST (BioAssaySystems#EASTR-100, Hayward, CA, USA) according to the manufacturer’s instructions.

### 2.9. Detection of Anti-GALNS Antibodies

The anti-GALNS antibody responses were analyzed using the previously modified ELISA protocol [33]. Plasma concentrations of anti-GALNS antibodies were determined by extrapolating the absorbance values from a calibration curve using standard monoclonal anti-GALNS antibodies (Custom-made clone 2F5F2, Creative Biolabs, Shirley, NY, USA).

### 2.10. Luminex Assay

Cytokine detection was performed in the plasma of WYRGRL-LV-treated mice using a Procartaplex mouse cytokine screening panel (17-plex, ThermoFisher #EPX170-26087-901, Waltham, MA, USA) according to the manufacturer’s instructions.

### 2.11. Pathology and Immunohistochemistry

Following 16 weeks post-infusion in MKC2 mice, the heart, liver, tibia, and knee joints were collected in 10% formalin. The heart and knee joint tissues were then fixed in a 2% paraformaldehyde and 4% glutaraldehyde solution, and 0.5-μm-thick sections were prepared using toluidine blue staining. Furthermore, the evaluation of lysosomal storage by light microscopy was performed as follows: Bone and heart pathologies were assessed in a double-blinded manner by assigning scores to vacuolization and columnar structures in the relevant tissues, ranging from 0 (best) to 3 (worst). Subsequently, statistical analysis was performed. To evaluate chondrocyte storage, we used ImageJ (NIH) software (version 1.54p). We measured the cell size after calibrating the microscope objective (40×) with a stage micrometer (with a known distance) to present the data in micrometers (μm). We selected and marked the perimeter of each chondrocyte only in the proliferative region of the growth plate to reflect cell size, with 100 cells per slide [33].

For immunohistochemistry (IHC), the liver and tibia were initially fixed in 10% formalin, then sectioned at a thickness of 5 µm for analysis. The levels of GALNS enzyme, KS, and procollagen were examined in both the tibia and liver by IHC using specific antibodies: anti-procollagen (Invitrogen#BTE0030202, Waltham, MA, USA), anti-KS (Santa Cruz Biotechnology#sc-73518, Dallas, TX, USA), and custom-made monoclonal anti-GALNS antibodies from Creative Biolabs, NY, USA [30,33]. IHC images were analyzed using ImageJ (NIH). Raw images were first converted to 8-bit grayscale to facilitate subsequent processing. Measurement parameters were set via the “Set Measurements” function, including area, mean gray value, and integrated density. Thresholding was performed with the “Adjust Threshold” tool to isolate positively stained regions. Quantification was carried out using the “Analyze” function. The results were obtained after excluding background noise and artifacts.

For HE staining, the whole bone (Tibia–joint–femur) of 1-day-old newborn MKC2 untreated and WT mice was fixed in 10% formalin. Since the bones of 1-day-old newborns were still composed of fully cartilaginous tissue, we did not undergo decalcification. After fixation and paraffin embedding, the samples were sectioned at a thickness of 5 µm for HE staining.

### 2.12. Bone Morphometric Analysis

A μCT scan was performed on the right femur of WT, MKC2, and LVGT groups using the SkyScan 1276 microCT System (Bruker, Manning Park, MA, USA), as described in our previous study [33].

### 2.13. Statistical Analysis

Quantitative data with a normal distribution were presented as mean ± standard error, while data without a normal distribution were reported as median (95% confidence interval). Shapiro–Wilk and Kolmogorov–Smirnov tests were conducted to determine whether the data followed a normal distribution. For statistical analysis involving more than two groups, one-way ANOVA with Tukey post hoc or Kruskal–Wallis test with Dunn’s multiple comparisons test was used, depending on the data’s distribution. A *p*-value of 0.05 or less was considered statistically significant. Two-way ANOVA was applied for analyses involving two variables. Pearson correlation analysis (two-tailed) determined the relationship between plasma enzyme activity and total anti-GALNS antibodies. Comparisons included: treatment vs. wild-type, treatment vs. untreated, and treatment vs. treatment. All statistical analyses were performed using GraphPad Prism 9.5.0 (GraphPad, San Diego, CA, USA). The number of independent biological replicates (*n*) was five for the in vivo experiments (*n* = 5).

## 3. Results

### 3.1. Pathology of Newborn MPS IVA Mouse Bone

**Figure 3:**
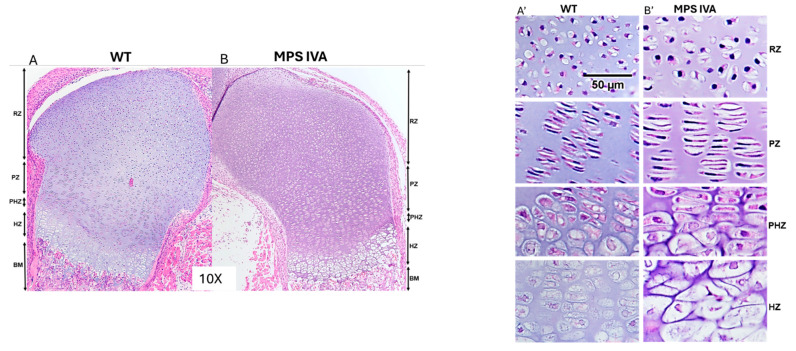
Bone in early development. HE staining of the right leg femurs from 1-day-old male mice at 10× (**A**,**B**) and 40× (**A’**,**B’**) magnification. The femurs shown were taken from the C57B6/6J background wild-type (**A**) and untreated MPS IVA (**B**) mouse models. RZ: Resting zone, PZ: Proliferative zone, PHZ: Proliferative–hypertrophy transition zone, HZ: Hypertrophic zone, BM: Bone marrow, WT: Wild-type.

To confirm the status of the growth plate zones in the MPS IVA mouse model, we stained the femoral cap of 1-day-old newborn mice with HE. The wild-type femur cap exhibited an organized columnar structure in the proliferative and hypertrophic zones, characterized by a high cell density. There was no secondary ossification center. The growth plate region in 1-day-old MPS IVA mice already contained ballooned vacuolated chondrocytes in the resting and proliferative zones (Figure 3). The cells in the hypertrophic zone were swollen, with increased fibrillary or vacuolar contents. The hypertrophic zone, although hypercellular, exhibited disorganization with a distorted cellular arrangement. We examined the femur, tibia, knee joint, and ankle junctions to determine the status of calcification in the femoral bones of 1-day-old newborn mice, confirming that no calcification had yet occurred in any of these areas. Importantly, the secondary ossification center had not developed yet in the epiphyseal plate of these newborn bones 1 day after birth (Figure 3A,B). These findings encouraged us to utilize IA injections as well in newborn mice to test the efficacy of collagen type II-targeting LV.

### 3.2. GALNS Enzyme Activities in Plasma and Tissues

**Figure 4:**
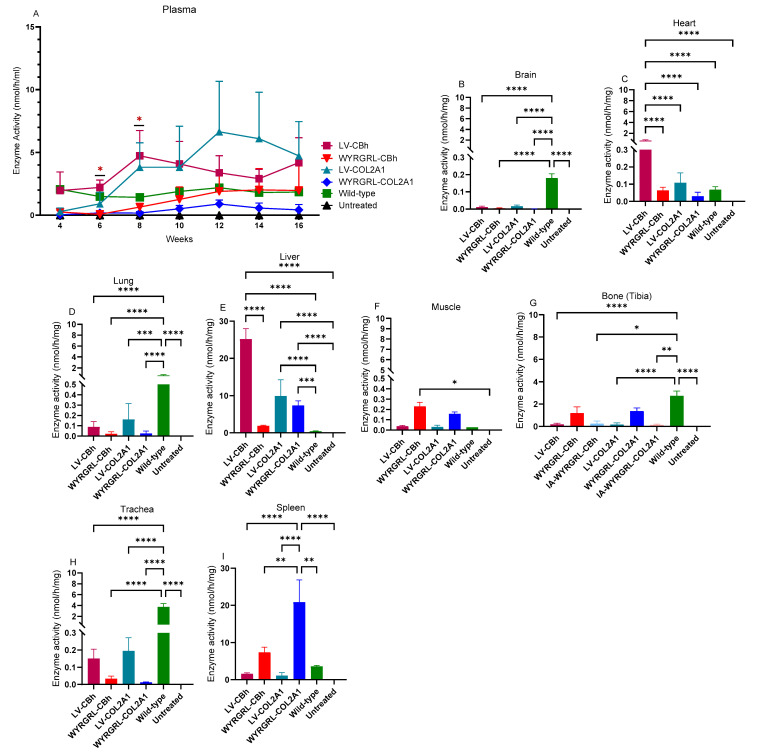
Enzyme activity at 16 weeks in tissues of mice treated with a 1 × 10^11^ TU/kg dose (**A**–**I**). (**A**) Plasma, (**B**) Brain, (**C**) Heart, (**D**) Lung, (**E**) Liver, (**F**) Muscle, (**G**) Bone (tibia), (**H**) Trachea, and (**I**) Spleen. One-way ANOVA with Tukey’s post hoc test is used for the brain, heart, lung, liver, muscle, bone (tibia), trachea, ear, xyphoid, lymph node, spleen, thymus, BMCs, and WBCs, while two-way ANOVA is applied for plasma enzyme activity. *: *p* < 0.05, **: *p* < 0.005, ***: *p* < 0.001, ****: *p* < 0.0001; LV-treated group vs. WYRGRL-LV-treated groups; LV-treated groups vs. wild-type group, and LV-treated groups vs. untreated group. All groups included at least 5 mice, except for the WYRGRL-LV-COL2A1 group, which had 4 mice in the group.

We conducted in vivo administration of WYRGRL-LVs into MPS IVA newborn mice, either IV or IA, on day 1. Our expression cassette was designed under the CBh or COL2A1 promoter encoding the native *GALNS* gene. In our previous study, we evaluated the efficiency of conventional VSVG-LVs with the CBh and COL2A1 promoters in different age groups of the same mouse model. We investigated a minimally effective dose to treat this skeletal disorder. Based on these findings, we infused WYRGRL-LVs at a dose of 1 × 10^11^ TU/kg to compare transduction efficiency and other downstream effects between conventional VSVG-LVs and collagen type II-targeting WYRGRL-LVs. WYRGRL-LVs achieved higher transduction efficiency than conventional VSVG-LVs under the same conditions, leading to increased GALNS enzyme levels. Systemic delivery demonstrated that WYRGRL-engineered LVs have better therapeutic effects than local administration, although WYRGRL-LVs did raise GALNS enzyme levels in bone through local delivery. Additionally, we measured GALNS enzyme activity in plasma samples collected biweekly and in autopsied tissue samples. WYRGRL-CBh and COL2A1 LVs gradually increased GALNS enzyme levels in plasma, reaching normal (wild-type) levels (Figure 4A). Both LVs showed similar patterns in plasma, indicating the same secretion levels after production. We observed increased GALNS enzyme activity in the heart, lung, liver, muscle, bone, trachea, ear, xiphoid, lymph nodes, spleen, thymus, bone marrow cells (BMCs), and white blood cells (WBCs) with WYRGRL-LVs (Figure 4C–I and Figure S1). The WYRGRL-COL2A1 had the highest enzyme activity in the liver and spleen (Figure 4E–I). Compared with conventional VSVG-LVs, which showed the highest activity in the liver (Figure 4E), the novel collagen type II-targeting LV exhibited lower enzyme activities in the liver, indicating possible detrimental effects resulting from the WYRGRL peptide addition. However, the secreted GALNS enzyme levels from both VSVG-LVs and WYRGRL-LVs were similar, showing no significant differences (Figure 4A). The bone was the primary target in this treatment, and its enzyme activity was elevated whenWYRGRL-LV were systemically infused compared to intra-articular (IA) infusion. Both injection sites confirmed an elevation of the enzyme activities in various tissues after treatment with WYRGRL-LVs.

### 3.3. Mono-Sulfated KS Levels in Plasma and Tissues

**Figure 5:**
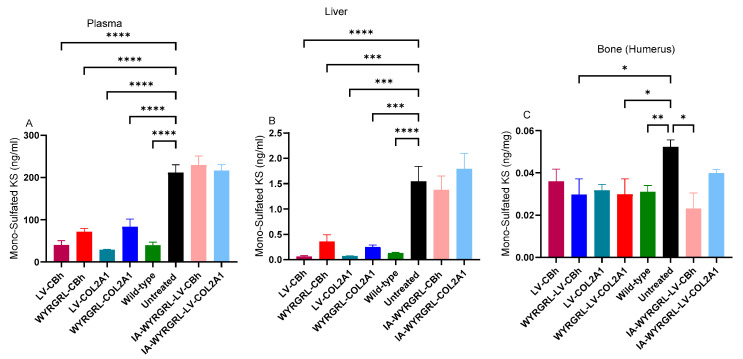
Mono-sulfated KS at 16 weeks. (**A**) Plasma, (**B**) Liver, and (**C**) Bone (humerus) of IV-injected mice treated with a dose of 1 × 10^11^ TU/kg. One-way ANOVA with Tukey’s post hoc test. Untreated group vs. LV-treated groups *: *p* < 0.05, **: *p* < 0.005, ***: *p* < 0.001, ****: *p* < 0.0001.

To confirm the therapeutic effectiveness of collagen type II-targeting WYRGRL-LVs, mono-sulfated KS levels were measured by LC-MS/MS in plasma, WBCs, BMCs, lung, liver, muscle, and humerus (Figure 5 and Figure S2).

All IV-injected WYRGRL-LVs significantly decreased mono-sulfated KS levels in the plasma, liver, and humerus. At the same time, there were no notable differences in KS levels in WBCs, BMCs, lungs, and muscle. Additionally, IA-injected WYRGRL-LVs confirmed reduced mono-sulfated KS levels in bone, with no change observed in plasma and liver, as expected from local treatment. No significant differences in tissue KS levels were found between the CBh and COL2A1 promoters with WYRGRL-LV. However, compared to VSVG-LVs, mono-sulfated KS levels were significantly lower in the bone after IV or IA injection of WYRGRL-LVs. Overall, collagen type II-targeting WYRGRL peptides led to a significant reduction in KS.

### 3.4. Distribution Patterns of LVs

**Figure 6:**
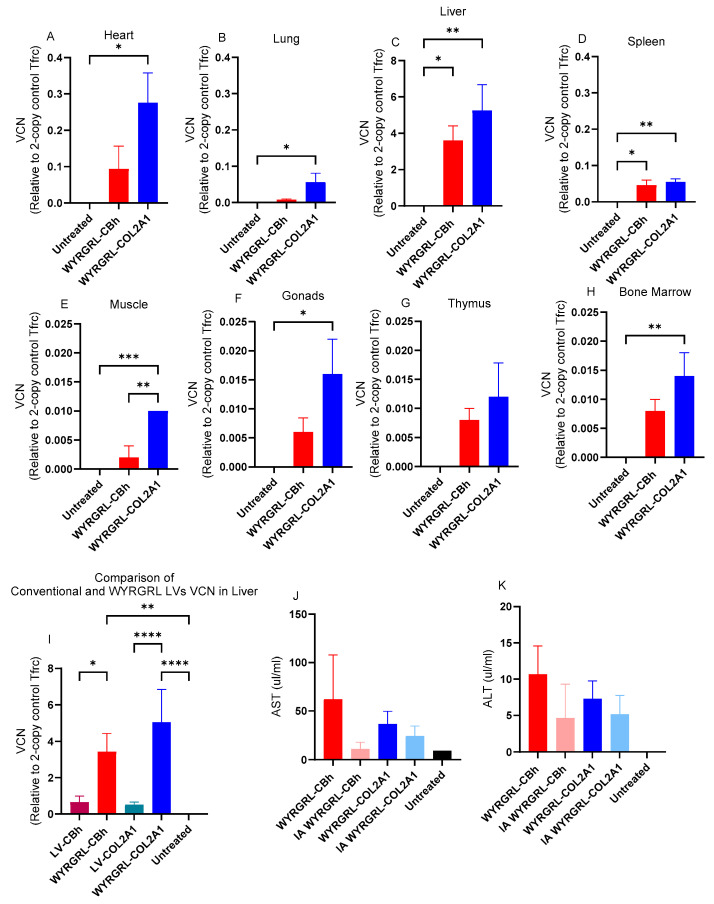
VCN 16 weeks post-infusion. (**A**) Heart, (**B**) Lung, (**C**) Liver, (**D**) Spleen, (**E**) Muscle, (**F**) Gonads, (**G**) Thymus, (**H**) Bone marrow, (**I**) Comparison of conventional and WYRGRL-LV VCN in the liver, (**J**) AST level, and (**K**) ALT level. One-way ANOVA with Tukey’s post hoc test and *t*-test is performed according to the normality of distribution determined using the Shapiro test. LVGT groups vs. untreated; *: *p* < 0.05, **: *p* < 0.005, ***: *p* < 0.001, ****: *p* < 0.0001.

Differences in GALNS enzyme levels between VSVG-LVs and WYRGRL-LVs raised the first key question: whether collagen-targeting WYRGRL peptide engineering influenced the viral properties or transgene expression compared to conventional VSVG-LVs. To answer this, we first evaluated VCN across all tissues, including the heart, lungs, liver, spleen, muscle, gonads, thymus, bone, and bone marrow, to confirm which tissues were effectively transduced and produced GALNS enzymes (Figure 6A–H). Then, we selected the liver with the highest VCN among other tissues from mice treated with WYRGRL-LVs to compare the VCN of liver samples treated with VSVG-LVs (Figure 6I). Except for the liver, all analyzed tissues showed very low or undetectable VCNs, but bone showed no VCNs. The liver was the most transduced tissue by WYRGRL-LVs, with an average of 3.6 ± 0.8 WYRGRL-CBh copies integrated into the genome. In contrast, WYRGRL-COL2A1 copies averaged 5.3 ± 1.4 viral copies per diploid cell (Figure 6C). Additionally, some individual mice treated with WYRGRL-COL2A1 had VCNs ranging from approximately 6 to 10 copies per diploid cell in the liver samples. We then compared these results with those from conventional VSVG-LVs (Figure 6I). This showed a 5.3-fold increase in VCN with WYRGRL-CBh and a 9.8-fold increase with WYRGRL-COL2A1 compared to conventional VSVG-LVs. Overall, collagen type II-targeting WYRGRL peptides enhanced LV uptake into the liver more than VSVG-LVs (Figure 6I). Consequently, most of the LVs were retained in the liver rather than delivered to the bone and cartilage. Additionally, there was no hepatocytic toxicity after WYRGRL-LVGT and conventional VSVG-LVGT administration (Figure 6J,K).

### 3.5. GALNS mRNA Expression After WYRGRGL-LVGT

**Figure 7:**
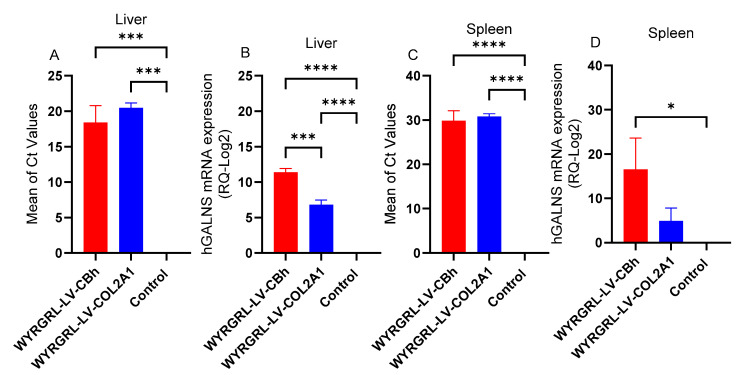
Analysis of *GALNS* mRNA in the liver of IV-treated mice with a 1 × 10^11^ TU/kg dose. (**A**,**B**). Liver and (**C**,**D**). Spleen. The graphs show the mean of amplification cycles (Ct) and *GALNS* mRNA expression. LVGT groups vs. untreated group *: *p* < 0.05, ***: *p* < 0.001, ****: *p* < 0.0001.

To confirm the expression of the *GALNS* gene driven by the CBh or COL2A1 promoter, we also analyzed *GALNS* mRNA levels in the liver, spleen, lung, ear, and bone 16 weeks after treatment. The number of amplification cycles (Ct) indicated the presence of the targeted sample. Both WYRGRL-LV-CBh and COL2A1 showed a high level of the target in the liver (Figure 7A). GALNS mRNA levels were calculated using the ΔΔCt method in RT-PCR. WYRGRL-LV-CBh-treated mice exhibited nearly a 12-fold increase in *GALNS* mRNA expression in the liver, while WYRGRL-LV-COL2A1 showed a 7-fold increase (Figure 7B).

In the spleen, we found that the mean Ct values for both LVs ranged from 30 to 40, indicating a lower concentration or even absence of the target (Figure 7C,D). Similarly, we could not detect mRNA expressions in the ear, bone, or lung, which may be related to low or undetectable RNA concentrations (<2 ng/μL).

### 3.6. Cytokine Analysis

**Figure 8:**
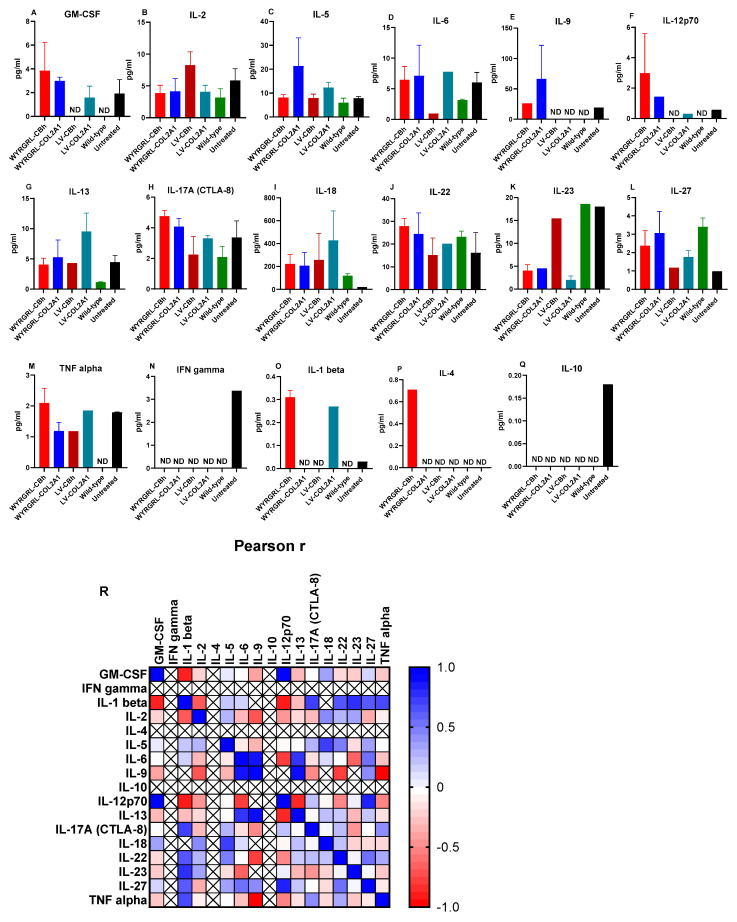
Cytokine analysis in plasma of MPS IVA mice after WYRGRL-LV gene therapy (**A**–**Q**). Kruskal–Wallis test with Dunn’s post hoc for non-normal distributions, while one-way ANOVA with Tukey’s test is used for normal distributions with more than two independent groups. No significant differences were found (*n* = 5). Some cytokines were detected in only one mouse out of the entire group because their levels were below the detection limit. They were not included in the analysis; IFN gamma, IL-1 beta, IL-4, IL-9, and IL-10 are some of them, with *n* = 1 or 2 mice. (**R**). Pearson correlation heatmap of cytokine assay results. The heatmap displays Pearson correlation coefficients among measured cytokines in the plasma of MPS IVA mice. Colors indicate the strength and direction of correlations, with red indicating a strong negative correlation (*r* = −1), blue indicating a strong positive correlation (*r* = +1), and white representing no correlation (*r* = 0). The intensity of the color corresponds to the magnitude of the correlation coefficient. Hierarchical clustering was applied to group cytokines with similar correlation patterns. ND: Not detectable—values below the lower limit of quantification. Values above the upper limit of quantification were capped unless reliable extrapolation was possible. No values were replaced with zero to prevent artificial suppression of analyte presence.

Cytokine analysis was conducted across all experimental groups (WYRGRL–LVs, LV alone, untreated MPS IVA, and wild–type controls) to evaluate immune activation and inflammatory responses related to lentiviral infusion. This helped us differentiate vector-related effects from disease-associated cytokine changes and to assess the safety of bone-targeting modifications. We quantified a panel of cytokines in plasma samples after 12 weeks of LV infusion and compared them with control groups. Although we did not find a statistically significant difference between WYRGRL-LVs and the other groups, important findings emerged in untreated MPS IVA and wild-type samples. We detected GM-CSF, IL-9, IL-12p70, TNF alpha, IFN gamma, IL-1 beta, and IL-10 in untreated MPS IVA mouse plasma, although wild-type groups did not show an elevation of such cytokines. After LV treatments, these cytokines were downregulated in MPS IVA mice. Non-significant differences between WYRGRL-LV and VSVG-LV did not indicate which LV was more effective. Conversely, compared to wild-type controls, there was a non-significant increase in specific cytokines in both LV-treated groups, including GM-CSF, IL-6, IL-9, IL-12p70, IL-17A (CTL-8), IL-22, IL-27, TNF alpha, IL-1 beta, and IL-4 in WYRGRL-LV-treated groups, and IL-2, IL-6, IL-13, IL-18, IL-23, TNF alpha, and IL-1 beta in LV-treated groups (Figure 8). Overall, the WYRGRL peptide did not trigger an unexpected cytokine release in the groups treated.

### 3.7. Anti-GALNS Antibodies

**Figure 9:**
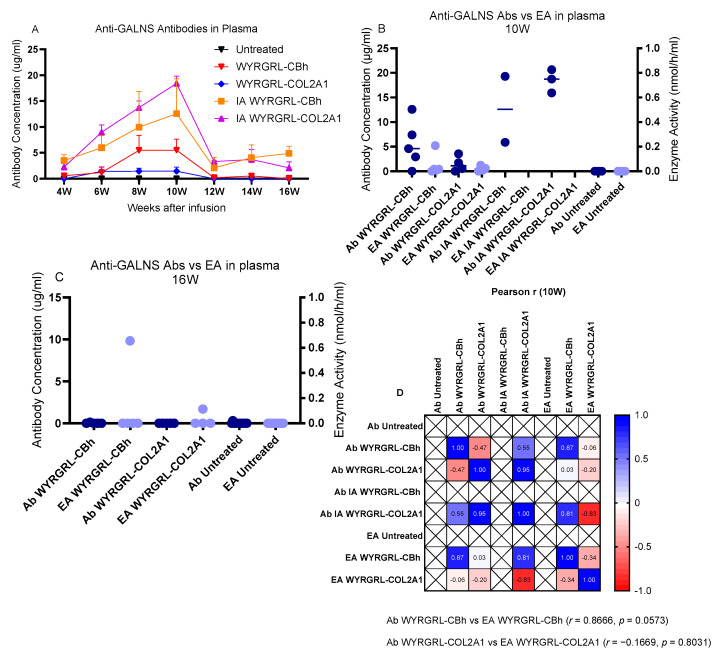
Evaluation of anti-GALNS antibodies. (**A**) Plasma anti-GALNS antibody levels over 16 weeks; two-way ANOVA with Tukey’s multiple comparison test. (**B**,**C**) Pearson correlation analysis (two-tailed) of plasma enzyme activity and total anti-GALNS antibodies at 10 and 16 weeks (EA: enzyme activity, Ab: antibody). (**D**) Pearson correlation heatmap of the correlation of the GALNS enzyme activity and anti-GALNS antibody results. Colors indicate the strength and direction of correlations, with red indicating a strong negative correlation (*r* = −1), blue indicating a strong positive correlation (*r* = +1), and white representing no correlation (*r* = 0). The intensity of the color corresponds to the magnitude of the correlation coefficient. Hierarchical clustering was applied to group cytokines with similar correlation patterns.

We performed ELISA on plasma samples to assess the immune response to the GALNS enzyme. Total anti-GALNS antibodies were elevated and peaked at 10 weeks, then declined and stabilized by 16 weeks (Figure 9A). We compared GALNS enzyme activity and anti-GALNS antibody levels between 10 and 16 weeks (Figure 9B,C). Since plasma enzyme activities and antibody levels were nearly zero, we created a correlation matrix for only week 10, which showed the highest antibody levels (Figure 9D). Although enzyme activity was negatively correlated with antibody levels in the WYRGRL-COL2A1 group (r = −0.1669), the increase in total anti-GALNS antibodies against the enzyme activities at 10 and 16 weeks was not significant(*p* = 0.8031).

### 3.8. A Slight Correction Was Detected in the Heart and Bone

**Figure 10:**
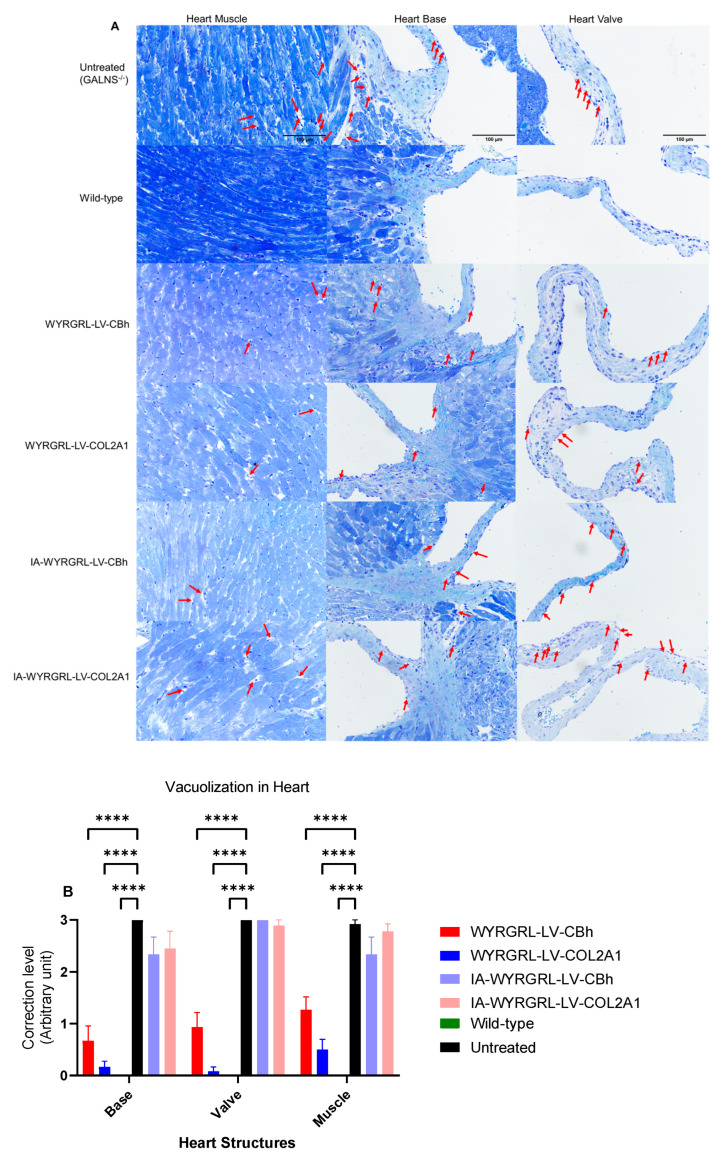
Heart pathology after lentiviral vector treatment. (**A**) The image shows the heart muscle, base, and valve at 40× magnification, with a 100-µm scale bar (red arrows for vacuolated cells). (**B**) Vacuolization in heart cells. Kruskal–Wallis test with Dunn’s multiple comparisons; LVGT groups vs. untreated group and LVGT groups vs. wild-type group: ****: *p* < 0.0001.

We evaluated the heart and bone for pathological improvement following IV or IA infusion of WYRGRL-LVs. In the heart, the valvular area, endocardial membrane, and heart muscles were examined for the presence of vacuolated cells. Compared with untreated heart samples, we observed a reduction in vacuolation in heart cells in the muscle, base, and valvular regions; however, this reduction was insufficient to fully correct the disease pathology in the heart relative to wild-type samples (Figure 10A,B, Table S3).

**Figure 11:**
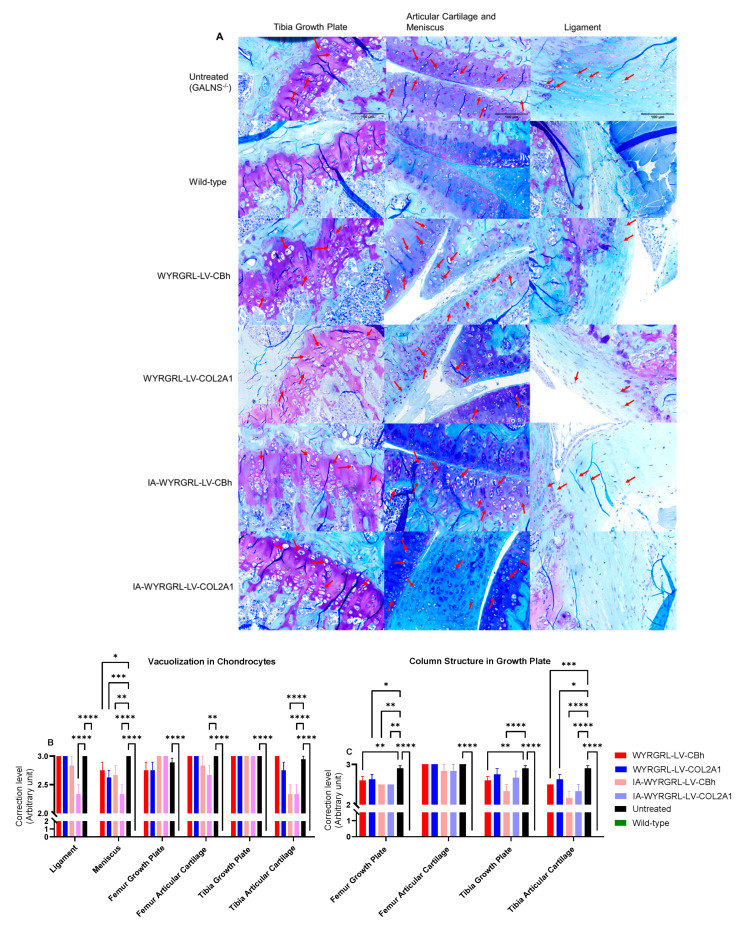

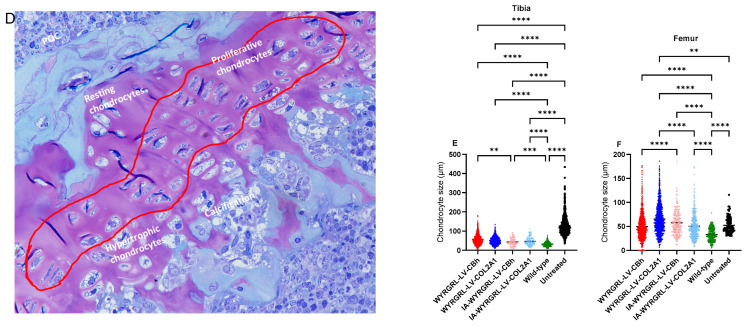
Bone pathology in lentiviral vector-treated and control groups. (**A**) The image shows the tibial growth plate, tibial and femoral articular cartilage, meniscus, and ligament at 40× magnification with a 100-μm scale. POC, Primary ossification center; SOC, Secondary ossification center; GP, Growth plate; (**A**,**C**) Articular cartilage; M, Meniscus; L, Ligament. Red arrows show the vacuolated cells. (**B**) Chondrocyte vacuolization. (**C**) Chondrocyte column structure. Since the wild-type group in (**B**,**C**) represented no vacuolization and an organized columnar structure, it was scored as zero. (**D**) Growth plate zones and evaluation area—proliferative zone. (**E**) Tibia chondrocyte size analysis. (**F**) Femur chondrocyte size analysis. Kruskal–Wallis with Dunn’s test. The average chondrocyte size is reported as the median, with a 95% confidence interval covering individual values. LVGT groups compared to the untreated group and the untreated vs. the wild-type group: *: *p* < 0.05, **: *p* < 0.005, ***: *p* < 0.001, ****: *p* < 0.0001.

In the bone, the articular cartilage, growth plates of the femur and tibia, ligaments, and menisci were evaluated for vacuolation in chondrocytes (Figure 11A,B, Table S3). Additionally, the growth plate area was assessed for the organization of the columnar structure (Figure 11A–C). We confirmed chondrocyte size by measuring the perimeter of each chondrocyte in the proliferative zones to verify reductions in storage materials (Figure 11D,E). Both IV and IA-infused groups showed partial correction in the targeted areas. IA-infused WYRGRL-LVs improved vacuolation in the articular cartilage, meniscus, and ligament more effectively than the IV administration groups (Figure 11B). In the tibia and femur, the columnar structure was somewhat organized (Figure 11C). None of these treatments reached the growth plate areas of the tibia and femur (Figure 11B). The size of chondrocytes in the proliferative region indicated a significant reduction in storage materials in the tibia and femur compared to chondrocytes of untreated mice (Figure 11D–F).

We further analyzed the bone morphometry of trabecular and cortical bones using micro-computed tomography (μCT); however, no significant differences were observed in either type of bone (Figure S3).

### 3.9. Immunohistochemical Staining Using Anti-GALNS, Anti-KS, and Anti-Collagen Antibodies

**Figure 12:**
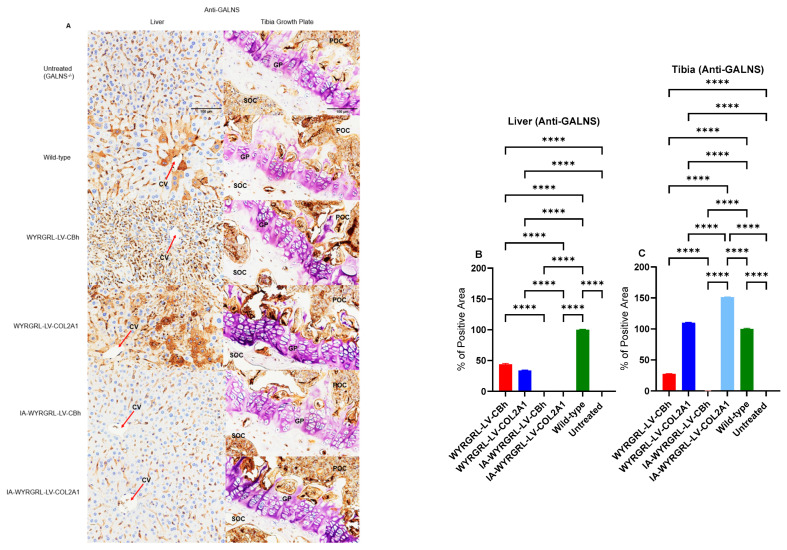
GALNS enzyme expression in liver and bone (tibia growth plate) under anti-GALNS staining; 40× magnification with a 100-µm scale. (**A**) POC, Primary ossification center; SOC, Secondary ossification center; GP, Growth plate (purple due to the presence of cartilage); and CV, Central vein (red arrows). Statistical analysis of expression levels in the liver (**B**) and tibia growth plate (**C**) is performed using one-way ANOVA with Tukey’s post hoc test. LVGT groups vs. the untreated group and LVGT groups vs. the wild-type group; ****: *p* < 0.0001.

We demonstrated the expression of GALNS enzymes through immunohistochemical analysis of the liver and tibia growth plate. To do this, the expression levels of GALNS enzymes and the subsequent reduction in KS and collagen levels were detected using specific antibodies, including anti-GALNS (Figure 12A–C), anti-KS (Figure S4B), and anti-collagen (Figure S4C). In the liver, GALNS enzyme expression significantly increased with IV treatment using WYRGRL-LV (~60%) compared to the untreated level (0%) (Figure 12A,B). The expression of the GALNS enzyme in the liver correlated with its activity there. In the bone, except for IA-injected WYRGRL-LV-CBh, all treatments confirmed GALNS enzyme expression in the proliferative area (Figure 12A–C). IA-injected WYRGRL-LV-COL2A1 showed the highest expression in bone compared to other LV treatments (Figure 12C). Although we assessed KS and collagen expression in both the liver and bone, we did not observe a significant reduction through IHC (Figure S4A,B).

### 3.10. Body Weight

We observed a slight increase in body weight in IV-treated mice; however, this increase was not statistically significant compared with untreated mice. Mice infused with IA showed no differences from the untreated group (Figure S5).

## 4. Discussion

This study is the first application of LVs modified with a collagen type II-targeting peptide to treat MPS IVA via in vivo direct infusions. We showed that adding the collagen type II-targeting WYRGRL ligand to the LV envelope increased the binding affinity to LVs in various tissues, including bone, cartilage, and other connective tissues, by IA injections. With this strategy, connective tissue cells such as chondrocytes, ligament cells, and meniscus cells in articular joint were exposed to higher concentrations of LVs, resulting in a higher transduction of LVs through both collagen and LDL receptors. As a result, GALNS enzyme activity was elevated at the target sites. On the other hand, MPS IVA is a systemic disease that requires delivering the GALNS enzyme to other tissues. Therefore, IV-infusion of the WYRGRL-LVs targets several tissues through LDL and other potential receptors or proteins and integrates their genomes into the cells. Consequently, the synthesized GALNS enzymes can be delivered systemically to other tissues following their secretion by the transduced cells.

Our experience with the efficiency of GALNS enzyme delivery alone suggests that reaching deep cartilage zones without tags or guides is quite limited. Penetration of GALNS enzymes into the cartilage is also restricted. Therefore, adding tags to GALNS enzymes can improve penetration and uptake into cartilage. Additionally, bone is a hard-to-reach tissue, and delivery of therapeutic agents into bone and cartilage is quite limited due to their avascularity. Delivering the produced enzyme directly into the bone and cartilage is challenging. Therefore, to deliver therapeutic agents to these tissues, ossification centers are required to develop. Primary ossification centers develop during early fetal life, while secondary ossification centers form after birth. The secondary ossification center is critical to target the articular cartilage and growth plate at early ages due to the presence of vasculature and bone marrow niche, although the deep zones of cartilage are still questionable [13,34,35]. Modifying the viral envelope with a cartilage-targeting peptide can elevate the enzyme activity. The WYRGRL peptide is a cell-targeting peptide with high affinity for cartilage, and it remains in mouse femoral cartilage for over 24 h. Furthermore, it can be trapped in the extracellular matrix (ECM), leading to high penetration into cartilage. However, our modified LVs could not reach the bone and cartilage to confirm the effectiveness of this strategy. As emphasized, the WYRGRL-LVs were unexpectedly trapped in the liver. This may be due to viral entry occurring via the liver ECM or some collagen receptors that share similar binding sequences. This could cause the lentivirus to be taken up by liver cells, rather than relying solely on VSVG tropism. We know that the liver does not have collagen type II, but other types, and the WYRGRL peptide provides adhesion to collagen type II. Therefore, this uptake may have also resulted from the unwanted adhesion of the virus to the cell membrane.

The expression of the *GALNS* gene driven by the CBh or COL2A1 promoter under the WYRGRL-LVs via in vivo direct IV administration was limited compared to our previous study with the conventional VSVG LVs [30]. Although this still requires further investigation, we assumed that the high integration of LV into the genome triggered a reduction in gene expression. We assumed that gene expression was impaired after integration. This assumption is based on the analysis of VCNs and biochemical assays relative to LVs without the WYRGRL peptide. WYRGRL–LVs were taken up by liver cells 5 to 10 times more than conventional LVs without the peptide (Figure 6I). These data showed opposite enzyme activities, with conventional LVs having higher activity in the liver than WYRGRL–LVs. Briefly, more viruses in the cells resulted in lower enzyme activities. In the context of LVs, random integration is a critical issue. If several LVs integrate into the genome simultaneously, it may cause unexpected events, as we experienced. However, AST/ALT did not indicate hepatic toxicity. As a result, we believe LV integration may not have affected genes related to cell fate or caused cellular stress. Of course, further investigation is needed to confirm this. The WYRGRL peptide is specific to collagen type II, which is abundant in cartilage because it is a precursor of endochondral ossification and suppresses chondrocyte hypertrophy [13,34]. Moreover, there is currently no information on the presence of collagen type II in the liver. Interestingly, inserting the WYRGRL peptide into VSVG may have altered its binding or trapping in the liver ECM via IV infusion. The WYRGRL peptide-guided nanoparticle targeted the extracellular matrix component of articular cartilage in osteoarthritis mice 72 times more effectively than the nanoparticle alone after IA [27]. This peptide has been successfully used to deliver nanoparticles, drugs, and extracellular vesicles to cartilage via local delivery [24,25,27]. We were unable to find clear information on the interaction between this peptide and liver cells. In conclusion, further investigation is necessary to determine the reasons for such liver capture.With this study, we increased the uptake of LVs using the same gene expression cassette that we previously investigated [29]. Due to the high VCNs in the cells, we believe that the ubiquitous CBh promoter negatively affected gene expression more than the COL2A1. This may indicate that more transcriptional factors might be involved in CBh at different integration sites, since COL2A1 is tissue–specific [35]. Although VCN was high in the liver, there were insufficient enzymes in circulation to correct the disease pathology. The main question here is how much of the produced enzymes were secreted from the cells. In plasma, we observed no differences among groups in GALNS enzyme activity. Although intracellular enzyme levels were low, secreted enzyme activities were similar. Therefore, we observed that KS levels were significantly reduced in the analyzed tissues. Plasma KS decreased due to the secreted GALNS enzymes in circulation. The liver produced GALNS enzymes and took them up from the bloodstream, and bone KS was cleared through cross–correction. We also confirmed these findings with GALNS mRNA expression in the liver, spleen, lung, ear, and bone. Except for the liver and spleen, the other tissues showed no mRNA expression. Considering the absence of VCN in the bone, all this data suggest that bone pathology was improved via cross–correction. Thus, the reduction of KS in bone results from cross–correction. Therefore, WYRGRL–LVs could not reach the cartilage and bone.

Viral titer is challenging to maintain in vivo LV gene therapies. Therefore, we investigated an approach that would not result in a low viral titer. Previous studies have shown a reduction in viral titer due to modifications of the viral envelope and complications with gene cassettes [36]. We also encountered the same problem while designing new lentiviral envelopes using different strategies. The VSVG protein on LV was modified with a single-chain variable fragment (scFv), and the viral titer remained unchanged [37]. In conclusion, the VSV glycoprotein can be modified rather than the lentiviral envelope. Adding the WYRGRL collagen type II targeting sequence into the N-terminus of VSVG did not affect viral assembly, resulting in no reduction in viral titer. This led us to administer the same dose that we identified as the minimum effective dose of IV-infused conventional LVs in MPS IVA mouse models.

Another critical aspect of effective gene therapies for MPS IVA is bone structure. We examined bone from MPS IVA newborn mice and confirmed minimal calcification at 1 day of age, although the timing of primary and secondary ossification center formation may differ between species (mouse vs. human). This is an important step in the early treatment of complex diseases. Currently, we believe the disease may have started even before birth, during fetal development. To determine the exact time when symptoms first appear, further studies are needed to enable prompt action. The absence of calcification on day 1 in MPS IVA mice can help target extracellular or chondrocyte receptors with ligands. IA injection of WYRGRL-LVs increased GALNS enzyme activity in the bone compared to IA injection of conventional VSVG-LVs. This confirms improved LV transduction via the WYRGRL-collagen type II interaction after administration to 1-day-old newborns. However, targeting deep-zone chondrocytes remains an unsolved mystery.

It remains a significant challenge to enhance the delivery of gene products to cartilage tissue. We will apply the bone-targeting WYRGRL-LV approach to ex vivo gene therapy and will test the WYRGRL-tagged GALNS LV construct to enhance cross-correction in target connective tissues.

## 5. Conclusions

The WYRGRL peptide binds to collagen type IIα1, improving cartilage targeting. However, engineering VSVG with the WYRGRL peptide could potentially redirect viral entry to different receptors. The success of transduction also depends on fusion activity and receptor-binding specificity. Several viral vectors can enter cells, but enzyme production and secretion might not be efficient. More research is needed to explore effective viral modifications and collagen-targeting strategies. Once these initial experiments identify the best conditions, we will move to large-scale preclinical trials, which will then lead to clinical applications.

## Data Availability

The original contributions presented in this study are included in the article/Supplementary Material. Further inquiries can be directed to the corresponding author.

## Supplementary Materials

The following supporting information can be downloaded at: https://www.mdpi.com/article/10.3390/cimb48010042/s1.

## Abbreviations

The following abbreviations are used in this manuscript:

MPS IVA	Mucopolysaccharidosis IVA
GALNS	N-acetylgalactosamine-6-sulfate sulfatase
GAGs	Glycosaminoglycans
KS	Keratan sulfate
C6S	Chondroitin-6-sulfate
LV	Lentiviral vectors
LVGT	Lentiviral gene therapy
VSVG	Vesicular stomatitis virus glycoprotein
IV	Intravenous
IA	Intraarticular
ERT	Enzyme replacement therapy
AAV	Adeno-associated virus
ECM	Extracellular matrix
HE	Hematoxylin and eosin
VCN	Vector copy number
LDL	Low-density lipoprotein
KO	Knockout
WBCs	White blood cells
BMCs	Bone marrow cells
LC-MS/MS	Liquid chromatography–tandem mass spectrometry
ddPCR	Digital droplet polymerase chain reaction
qPCR	Quantitative polymerase chain reaction
ALT	Alanine aminotransferase
AST	Aspartate aminotransferase
ELISA	Enzyme-linked immunosorbent assay
WT	Wild-type
UT	Untreated
IHC	Immunohistochemistry
μCT	Micro-computational tomography
