# Supplementary material for: Collagen Type II-Targeting Lentiviral Gene Therapy for Mucopolysaccharidosis IVA
Source: Curr Issues Mol Biol. 2025 Dec 27;48(1):42. doi: 10.3390/cimb48010042 (PMC12839894; doi:10.3390/cimb48010042)
Supplement: Supplementary file 1 [file cimb-48-00042-s001.zip › cimb-3968795-supplementary.pdf]

Supplementary data

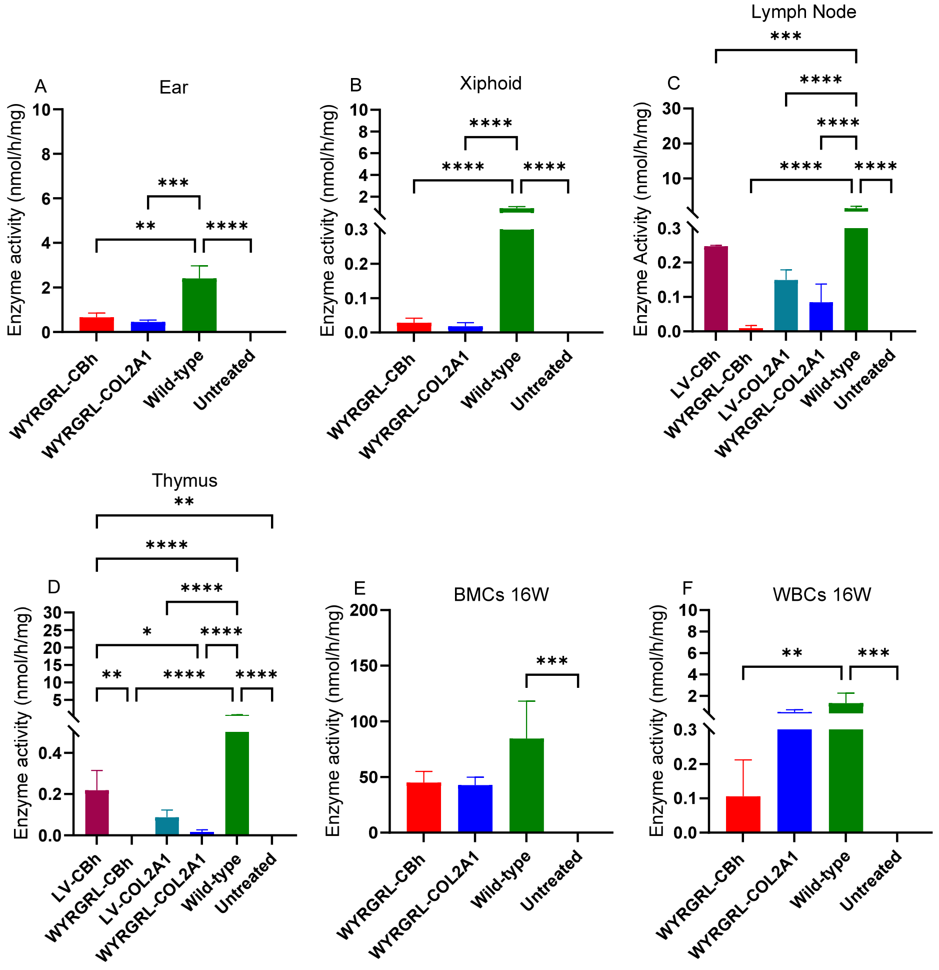

Figure S1. Enzyme activity at 16 weeks in tissues of mice treated with a 1 × 10[11] TU/kg dose **(A-O)**. **A.** Ear, **B.** Xyphoid, **C.** Lymph nodes, **D.** Thymus, **E.** BMCs, and **F.** WBCs. One-way ANOVA with Tukey's post-hoc test for the ear, xyphoid, lymph node, thymus, BMCs, and WBCs; *: < 0.05, **: < 0.005, ***: < 0.001, ****: < 0.0001; LV-treated group vs WYRGRL-LV-treated groups; LV-Treated groups vs. Wild-type group and LV-Treated groups vs. Untreated group. All groups had animal numbers equal to/more than 5 mice, except for the WYRGRL-LV-COL2A1, which had 4 mice in the group.

.

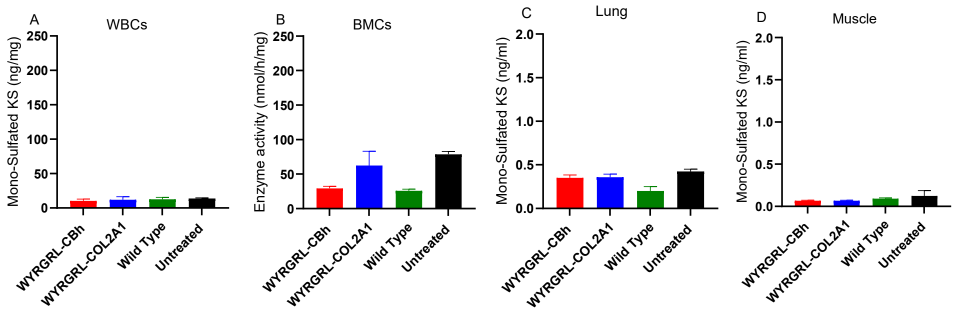

Figure S2. Mono-sulfated KS concentrations after WYRGRL-LVGTs at 16 weeks. **A.** WBCs, **B.** BMCs, **C.** Lung, and **D.** Muscle. LVGT groups vs. a wild-type group – not significant.

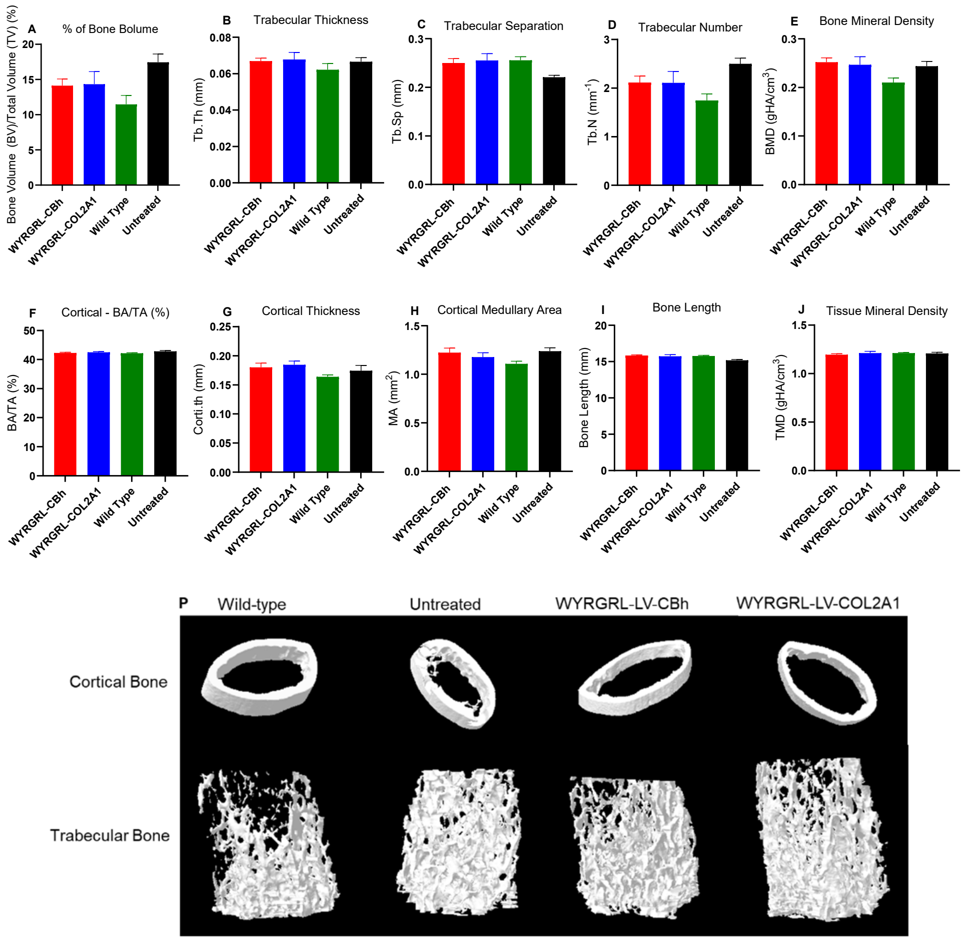

Figure S3. Bone morphometric analysis. Trabecular bone morphology **(A-E, P)** and cortical bone morphology **(F-J, P)**. One-way ANOVA with Tukey's post-hoc test. Treatment groups vs. an untreated group; Treatment groups vs. a wild-type group. No significance was found.

Table S1. Primers and probes for VCN analysis.

Primers	Sequences
LV-Psi-F (900 nM)	5′ -CGACTGGTGAGTACGCCAAA-3′
LV-Psi-R (900 nM)	5′ -CCCGCTTAATACTGACGCTCTC-3′
LV-Psi-Probe (250 nM)	5'-/6-FAM/AGCGGAGGC/ZEN/TAGAAGGAGAGAGATGGGT/IABkFQ/-3'
Amplicon Size	82 bp

Table S2. Heart pathology at 16 weeks. Levels of storage materials were scored. "No storage or very slight" was 0 (−), "slight but obvious" was 1 (+), "moderate" was 2 (++), and "marked" was 3 (+++). Each pathological slide was assessed in a double-blinded manner three times. *n* = 3-6. Data are presented as Mean ± SEM.

**Vacuolization**	**UT**	**WT**	**WYRGRL-LV-CBh**	**WYRGRL-LV-COL2A1**	**IA-WYRGRL-LV-CBh**	**IA-WYRGRL-LV-COL2A1**
Base	3.0±0.0	0.0±0.0	0.6±0.3	0.17±0.1	2.3±0.3	2.4±0.3
Valve	3.0±0.0	0.0±0.0	0.9±0.3	0.08±0.08	3.0±0.0	2.9±0.1
Muscle	2.9±0.08	0.0±0.0	1.3±0.2	0.5±0.2	2.3±0.3	2.7±0.1

Table S3. Bone pathology at 16 weeks. Levels of storage materials and degrees of disoriented columns were scored. "No storage or very slight" was 0 (−), "slight but obvious" was 1 (+), "moderate" was 2 (++), and "marked" was 3 (+++). Each pathological slide was assessed in a double-blind manner three times. *n* = 3-6. Data are presented as Mean ± SEM.

**Vacuolization**	**UT**	**WT**	**WYRGRL-LV-CBh**	**WYRGRL-LV-COL2A1**	**IA-WYRGRL-LV-CBh**	**IA-WYRGRL-LV-COL2A1**
Ligament	3.0±0.0	0.0±0.0	3.0±0.0	3.0±0.0	2.8±0.16	2.3±0.16
Meniscus	3.0±0.0	0.0±0.0	2.7±0.1	2.6±0.1	2.6±0.16	2.3±0.16
Femur Growth Plate	2.9±0.07	0.0±0.0	2.7±0.1	2.7±0.1	3.0±0.0	3.0±0.0
Femur Articular Cartilage	3.0±0.0	0.0±0.0	3.0±0.0	3.0±0.0	2.8±0.16	2.6±0.16
Tibia Growth Plate	3.0±0.0	0.0±0.0	3.0±0.0	3.0±0.0	3.0±0.0	3.0±0.0
Tibia Articular Cartilage	2.9±0.05	0.0±0.0	3.0±0.0	2.7±0.1	2.3±0.16	2.3±0.16
**Colum Structure**						
Femur Growth Plate	2.9±0.07	0.0±0.0	2.6±0.1	2.6±0.1	2.5±0.0	2.5±0.0
Femur Articular Cartilage	3.0±0.0	0.0±0.0	3.0±0.0	3.0±0.0	2.8±0.2	2.8±0.2
Tibia Growth Plate	2.9±0.07	0.0±0.0	2.6±0.1	2.7±0.1	2.3±0.2	2.6±0.2
Tibia Articular Cartilage	2.9±0.07	0.0±0.0	2.5±0.0	2.6±0.1	2.1±0.2	2.3±0.2

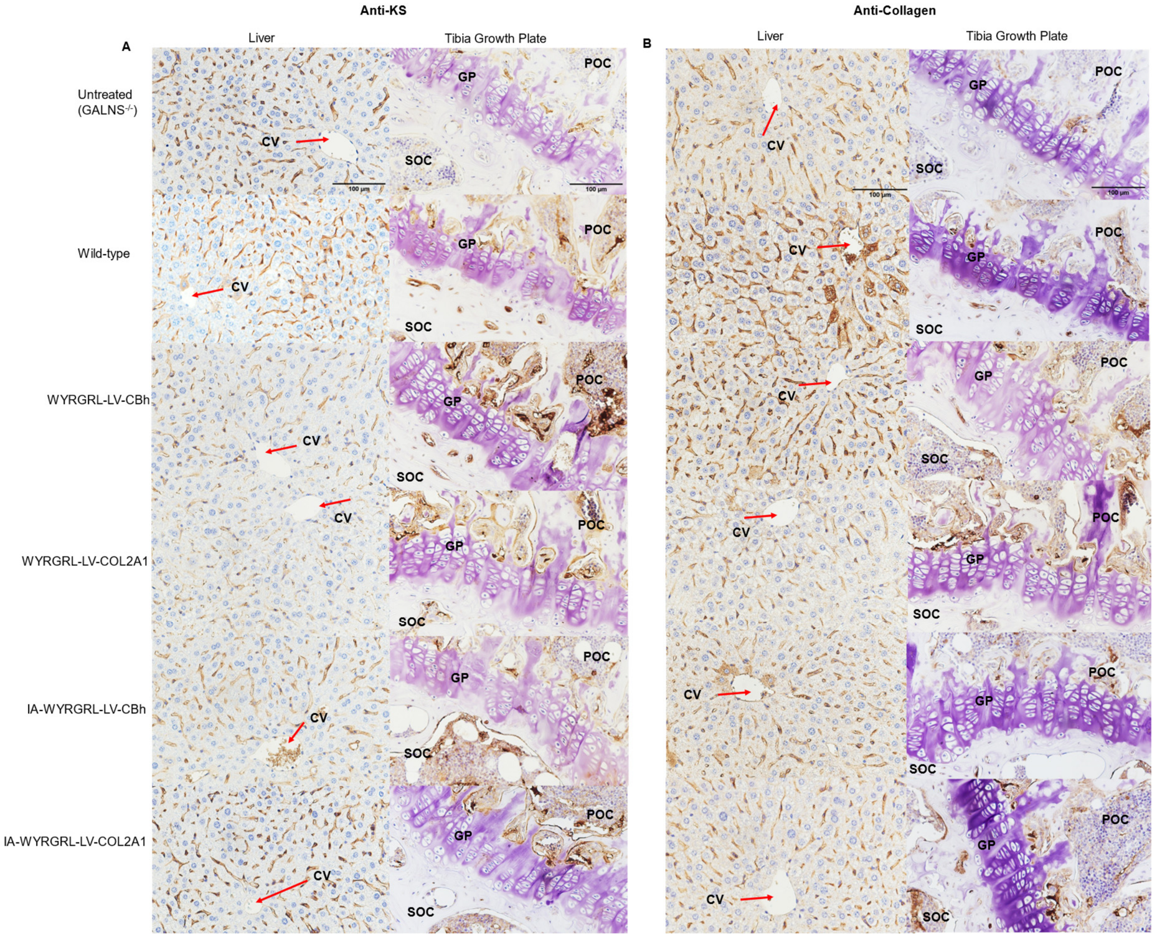

Figure S4. Anti-KS **(A)** and anti-collagen **(B)** staining of the liver and tibia growth plate at 40× magnification with a 100-µm scale. POC, Primary ossification center; SOC, Secondary ossification center; GP, Growth plate (purple); and CV, Central vein (red arrows).

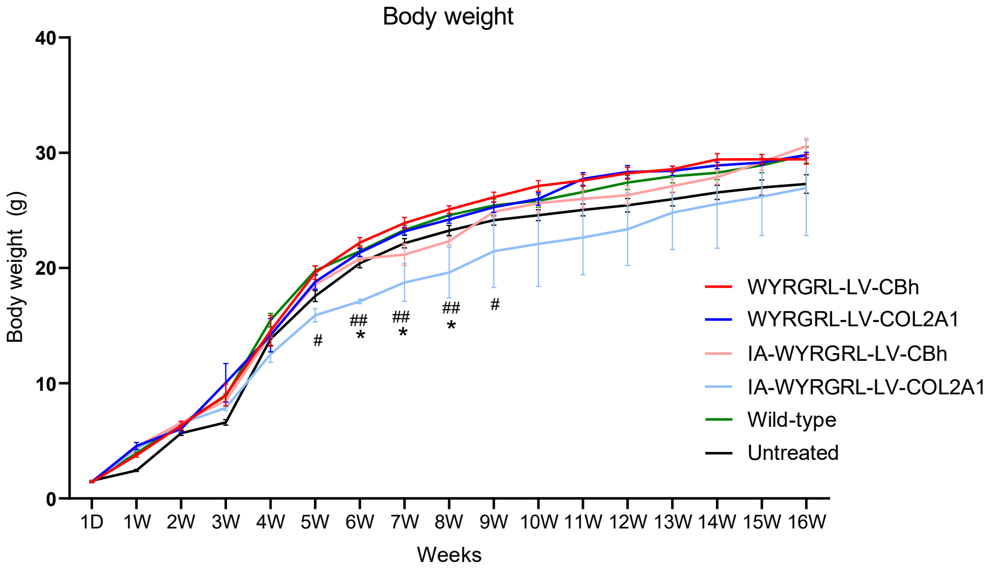

Figure S5. Bodyweight following 16 weeks after LVGT. 0 (Zero) represents a 1-day-old. Results were compared using one-way ANOVA with Tukey's post-hoc test as follows: LVGT groups vs. an untreated group; *: $p < 0.05$; LVGT groups vs. a wild-type group; #: $p < 0.05$, ##: $p < 0.005$.
